# The Consequences of Assisted Reproduction Technologies on the Offspring Health Throughout Life: A Placental Contribution

**DOI:** 10.3389/fcell.2022.906240

**Published:** 2022-05-20

**Authors:** Mariana Schroeder, Gina Badini, Amanda N. Sferruzzi-Perri, Christiane Albrecht

**Affiliations:** ^1^ Faculty of Medicine, Institute of Biochemistry and Molecular Medicine, University of Bern, Bern, Switzerland; ^2^ Centre for Trophoblast Research, Department of Physiology, Development, and Neuroscience, University of Cambridge, Cambridge, United Kingdom

**Keywords:** assisted reproductive technologies, placenta, epigenetics, metabolism, long-term health, DOHaD, fetal programming

## Abstract

The use of assisted reproductive technologies (ART) worldwide has led to the conception and birth of over eight million babies since being implemented in 1978. ART use is currently on the rise, given growing infertility and the increase in conception age among men and women in industrialized countries. Though obstetric and perinatal outcomes have improved over the years, pregnancies achieved by ART still bear increased risks for the mother and the unborn child. Moreover, given that the first generation of ART offspring is now only reaching their forties, the long-term effects of ART are currently unknown. This is important, as there is a wealth of data showing that life-long health can be predetermined by poor conditions during intrauterine development, including irregularities in the structure and functioning of the placenta. In the current review, we aim to summarize the latest available findings examining the effects of ART on the cardiometabolic, cognitive/neurodevelopmental, and behavioral outcomes in the perinatal period, childhood and adolescence/adulthood; and to examine placental intrinsic factors that may contribute to the developmental outcomes of ART offspring. Altogether, the latest knowledge about life outcomes beyond adolescence for those conceived by ART appears to suggest a better long-term outcome than previously predicted. There are also changes in placenta structure and functional capacity with ART. However, more work in this area is critically required, since the potential consequences of ART may still emerge as the offspring gets older. In addition, knowledge of the placenta may help to foresee and mitigate any adverse outcomes in the offspring.

## Introduction

Assisted reproductive technologies (ART) are increasingly used worldwide, to help couples conceive a child, most notably in Europe, where the largest number of ART treatments are performed (De Geyter, 2018; De Geyter et al., 2020). It is estimated that between 1978 - when the first *in vitro* fertilized child was born - and 2018, over eight million babies were born following ART worldwide (De Geyter, 2018; Sunderam et al., 2019). Though obstetric and perinatal outcomes have improved over the years through the advent of single embryo transfer (Hoyos and Ory, 2021), pregnancies achieved by ART still appear to bear increased risks for the mother and the unborn child (rewiewed in Qin et al., 2015, 2016). Whether the additional maternal and neonatal risks are due to the technology itself or underlying infertility-linked factors remains unresolved (Hoyos and Ory, 2021). When the obstetric and perinatal outcomes of ART and spontaneously conceived (SC) pregnancies were compared using the same mother as control, the results were similar (Ganer Herman et al., 2021), suggesting that health outcomes may be predominantly linked to the parents and not necessarily to ART (Hwang et al., 2018; Molinaro, 2021).

**FIGURE 1:**
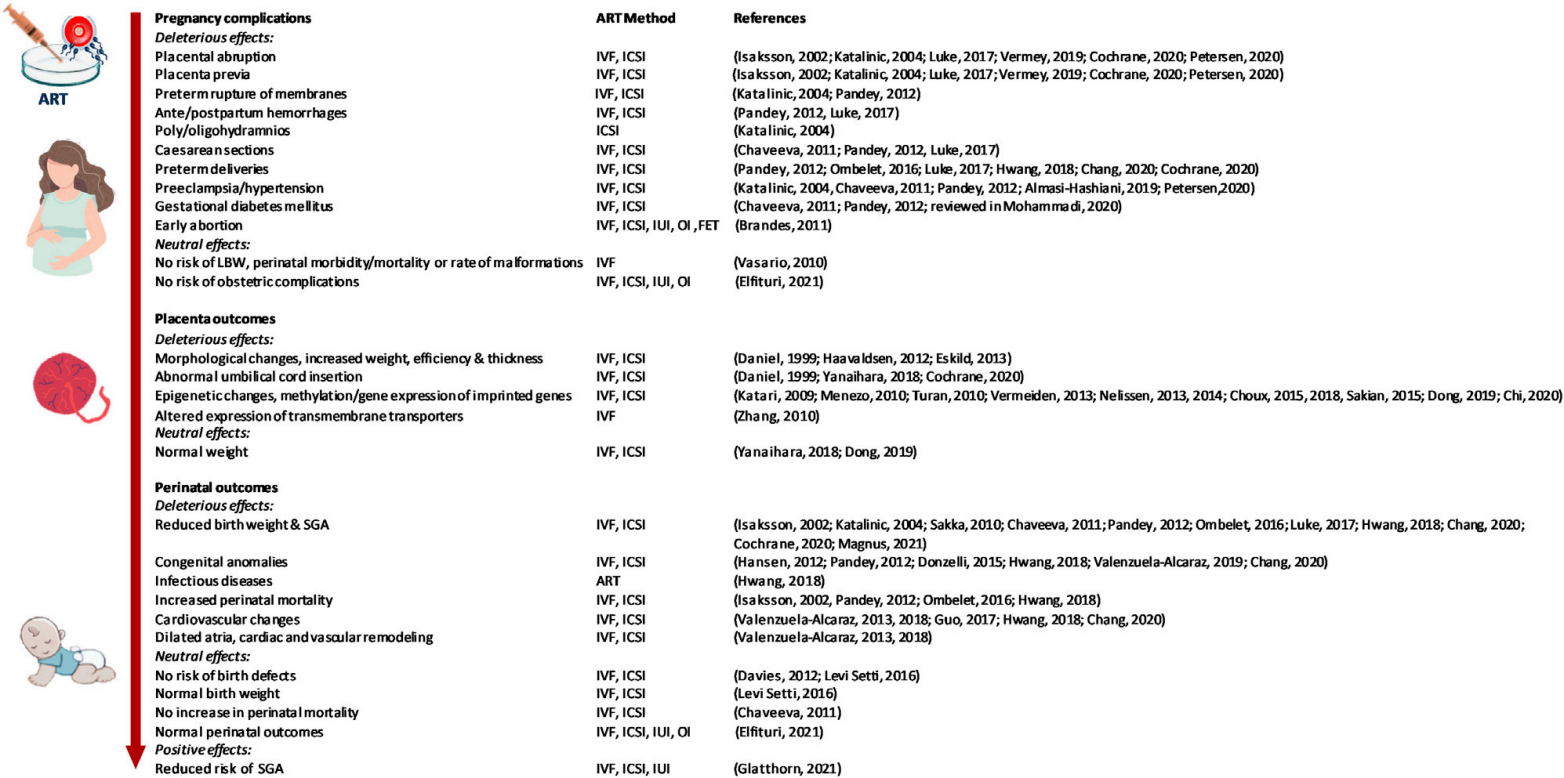
The effect of the use of ART on pregnancy complications, placental and fetal adaptations, and perinatal health outcomes. Legend: SGA: Small for Gestational Age. FET: Frozen Embryo Transfer. IUI: Intra-Uterine Insemination, OI: Ovulation Induction with Clomiphene citrate.

The phenomenon of an adverse *in utero* environment leading to the development of diseases later in life is well-known and referred to as the “developmental origin of health and disease” hypothesis. This states that the fetus undergoes adaptive changes to maintain homeostasis and to prepare the body for postnatal life. These adaptations depend on numerous factors, such as the type, length, and timing of the insult. Depending on their specific developmental windows, some organs may be programmed differently than others (Barker, 2012). To date, many conditions are believed to lead to fetal programming, including maternal under/overnutrition, smoking, physical inactivity, and psychological stress (Ravelli et al., 1999; Fleming et al., 2018; Lahti-Pulkkinen et al., 2018), preterm birth (Pandey et al., 2012; Qin et al., 2015; Ombelet et al., 2016; Qin et al., 2016; Hwang et al., 2018; Chang et al., 2020; Cochrane et al., 2020), gestational diabetes mellitus (GDM) (Chaveeva et al., 2011; Pandey et al., 2012; Qin et al., 2015, 2016; Mohammadi et al., 2020), preeclampsia (Chaveeva et al., 2011; Pandey et al., 2012; Qin et al., 2015, 2016; Almasi-Hashiani et al., 2019; Petersen et al., 2020), and infections (Fleming et al., 2018; Hwang et al., 2018; Lahti-Pulkkinen et al., 2018) (Figure 1)*.* Gestational insults lead to a 2–10-fold increase in the susceptibility to cardiovascular disease (CVD), type-2 diabetes mellitus (T2DM), obesity, cognitive dysfunction, and developmental disorders (e.g., autism, Asperger’s, and Rett’s syndromes) (Godfrey and Barker, 2000; Jirtle and Skinner, 2007; Van Den Bergh, 2011; Reynolds and Caton, 2012). In addition, heritable environmentally-induced epigenetic modifications resulting from gestational insults may also be transmitted across generations (Jirtle and Skinner, 2007; Nilsson et al., 2018; Beck et al., 2022).

While the exact mechanisms underlying developmental programming for each disease are yet to be elucidated, the placenta is believed to play a crucial role in this process (Reynolds and Caton, 2012). Placental nutrient and oxygen supply are vital for fetal development, thus an alteration in placental structure or function is of key relevance for fetal programming. Under adverse environmental conditions, the placenta undergoes adaptive changes to optimize nutrient and oxygen transport to the fetus (Burton et al., 2016). Such adaptions include changes in placental blood flow, morphology, transporter expression, and alterations in the epigenetic profile (Barker et al., 2010a, 2010b, 2012; Eriksson et al., 2011; Barker and Thornburg, 2013). These may, in turn, be exacerbated in pregnancies achieved through ART, considering the increased risks for pregnancy complications compared to SC pregnancies, and that pregnancies achieved by ART imply more stress, due to the many procedures the mother and fetus endure (Lahti-Pulkkinen et al., 2018).

In the current review, we aim to summarize the available knowledge on the effects of ART on offspring cardiometabolic, cognitive/neurodevelopmental, and behavioral outcomes, and to examine placental morphological, functional and epigenetic factors that may contribute to the developmental outcomes of ART offspring in humans. ART includes procedures like *in vitro* fertilization (IVF), intra-cytoplasmatic sperm injection (ICSI), surgical sperm retrieval, ovarian hyperstimulation, embryo culture, and embryo freezing. In the current review, we will focus on data reporting the effects of IVF and ICSI, as these are the most used.

## Perinatal Outcome in Newborns Conceived by ART

**FIGURE 2:**
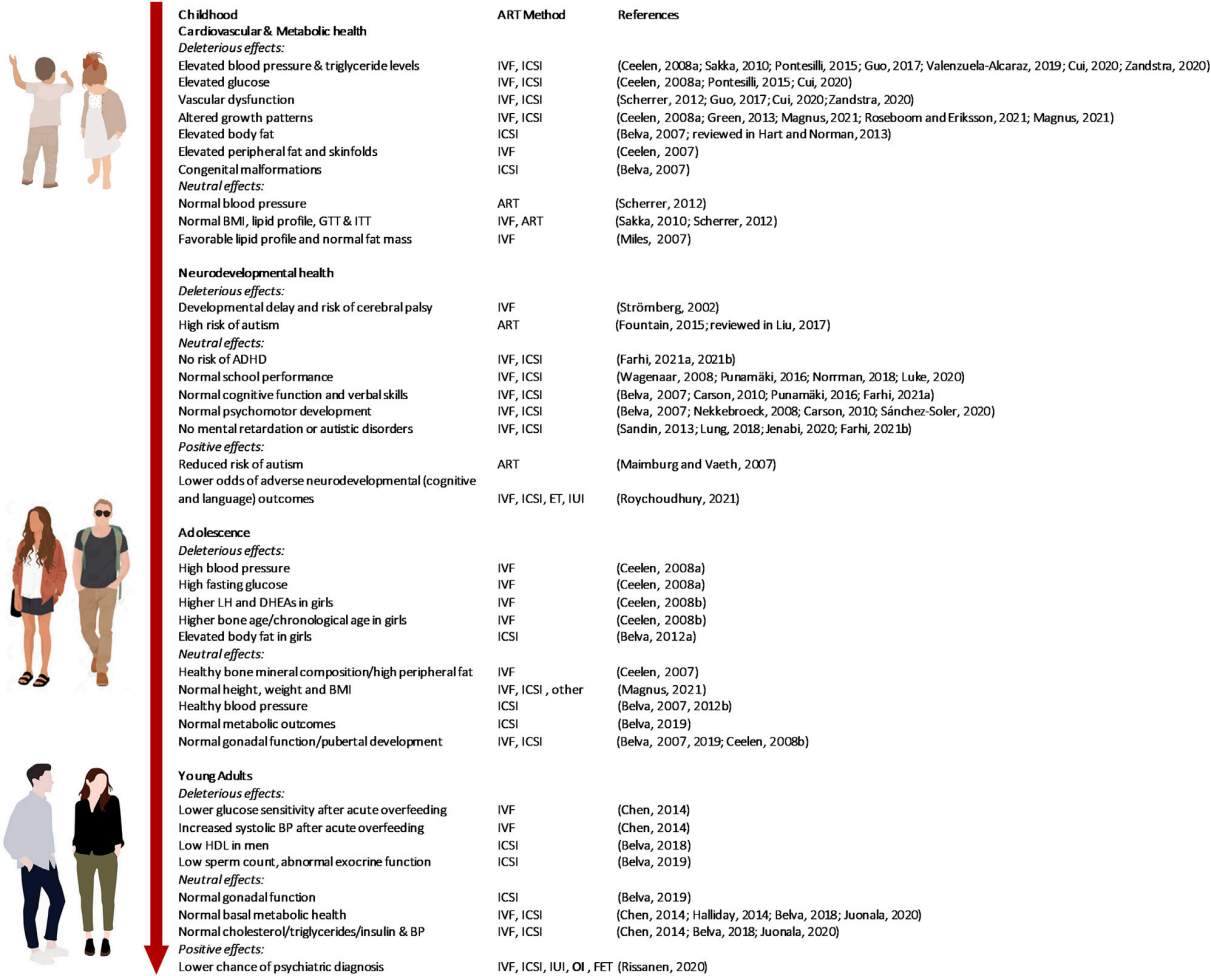
The effect of the use of ART on childhood, adolescent and adult health outcomes. Legend: LH: Luteinizing Hormone; DHEAS: Dehydroepiandrosterone sulfate; HDL: High-density lipoprotein; ADHD: Attention-deficit/hyperactivity disorder; BP: Blood pressure; BMI: Body Mass Index, GTT: Glucose Tolerance Test, ITT: Insulin Tolerance Test, ET: Embryo Transfer.

Many studies have investigated pregnancy outcomes, as well as short- (i.e. perinatal) and long-term (early life, adolescence, and young adulthood) outcomes in people born after ART procedures. Although most children born through ART are healthy, conception by ART has been linked to a variety of health complications and conflicting reports exist (See Figures 1, 2). For example, an increased risk for adverse perinatal outcomes including low birth weight (LBW) and small for gestational age (SGA) was shown when compared to SC pregnancies (Chaveeva et al., 2011; Pandey et al., 2012; Ombelet et al., 2016; Luke et al., 2017; Hwang et al., 2018; Chang et al., 2020; Cochrane et al., 2020). In contrast, a recent report found no differences and even reduced risk of SGA in ART offspring (Glatthorn et al., 2021). Findings pertaining birth weight were usually analyzed including the maternal weight/height as a covariate. Congenital anomalies also appear to be more prevalent in ART offspring compared to SC pregnancies (Hansen et al., 2012; Donzelli et al., 2015; Qin et al., 2016; Hwang et al., 2018; Valenzuela-Alcaraz et al., 2019; Chang et al., 2020), most notably for the cardiovascular (Valenzuela-Alcaraz et al., 2013, 2018, 2019), gastrointestinal and central nervous systems (Qin et al., 2015; Chang et al., 2020). Importantly, further studies reporting on birth defects showed these were linked to underlying infertility and not necessarily to ART, since children born to sub-fertile parents show similar health outcomes compared to ART conceived offspring (Davies et al., 2012; Levi Setti et al., 2016; Hwang et al., 2018). Finally, it has been suggested that ART may lead to an increased risk of cancer in the offspring, specifically for hematological malignancies, leukemia, neural and hepatic tumors (Hargreave et al., 2013; Wang et al., 2019). However, further studies showed no increased risk of cancer (Raimondi et al., 2005; Levi-Setti and Patrizio, 2018).

## Metabolic and Cardiovascular Health of Children Conceived by ART

The cardiovascular changes observed among ART-conceived children starting at the age of 3 years include dilated atria, more globular ventricles, endothelial dysfunction, signs of systolic and diastolic dysfunction, and systemic and pulmonary hypertension (Sakka et al., 2010; Zhou et al., 2014; Liu et al., 2015; von Arx et al., 2015; Guo et al., 2017; Valenzuela-Alcaraz et al., 2019; Zandstra et al., 2020). At the age of 6–10 years, ART offspring also presented a higher risk for metabolic dysfunction, with elevated fasting glucose, insulin, and insulin resistance compared to SC children (Chen et al., 2014; Pontesilli et al., 2015; Guo et al., 2017; Cui et al., 2020). They also presented elevated body fat and skinfolds (Ceelen et al., 2007; Hart and Norman, 2013). In contrast, Scherrer *et al* reported no differences in lipid profile, basal glucose, glucose and insulin tolerance, blood pressure, or body mass index (BMI) in ART children compared to SC controls. However, they found general and pulmonary vascular dysfunction in a cohort of 11-year-old children born through ART (Scherrer et al., 2012). In addition, despite presenting similar gross body size, the growth patterns of ART children appear strikingly similar to those of SC children who develop T2DM and CVD later in life (Barker et al., 2005; Magnus et al., 2021; Roseboom and Eriksson, 2021). These adverse outcomes persist when looking at singleton ART pregnancies only and thus, cannot be explained by multiple pregnancies after the use of ART (Pandey et al., 2012; Qin et al., 2015; Ombelet et al., 2016; Qin et al., 2016) (Figure 2).

## Cognitive and Neurodevelopmental Health of Newborns and Children Conceived by ART

ART children show similar neurological and psychomotor developmental faculties up to 3 years of age even when born preterm (Roychoudhury et al., 2021) compared with SC children (Nekkebroeck et al., 2008; Carson et al., 2010; Sánchez-Soler et al., 2020). Moreover, singletons and twins of both sexes conceived by ART perform as well as SC children once they reach school (Wagenaar et al., 2008; Punamäki et al., 2016; Norrman et al., 2018; Luke et al., 2020). Cognitive function, visual-motor ability, attention, and verbal skills of ART children were similar to SC children (Farhi et al., 2021a). Some reports suggested that ART does exert some negative influences on cognitive development. One study found a 4-fold higher risk of suspected developmental delay and cerebral palsy in IVF versus SC singletons. However, the effect disappeared when only twins were taken into account (Strömberg et al., 2002). Another study found a significant increase in the risk of mental retardation in ART children during their first year of life, but this association disappeared when the analysis was restricted to singletons (Sandin et al., 2013). There have been broad discussions about whether the use of ART is associated with the diagnosis of autism spectrum disorders (ASD) in the offspring. While most studies were unable to find any associations (Sandin et al., 2013; Lung et al., 2018; Jenabi et al., 2020; Farhi et al., 2021b), a few studies reported a significantly higher risk for ASD in ART offspring (Fountain et al., 2015; reviewed in Liu et al., 2017). However, the effect was substantially reduced when adverse prenatal and perinatal outcomes and demographics were taken into account (Fountain et al., 2015). Remarkably, a further study reported that ART children had a considerably lower risk of developing infantile autism compared to SC children (Maimburg and Vaeth, 2007). Altogether, the mental and sensory health outcomes and communication skills of children born through ART are reassuring and like that from SC pregnancies (Figure 2).

## Effects of ART on Long-Term Health (Adolescents and Young Adults)

Given that the first generation of ART children is now only reaching their early forties, there are, to date, no studies investigating health outcomes in adults of older age, i.e., when developmental programming would be expected to manifest more robustly. However, a few recent studies reporting on the health of adolescents and young adults suggest the long-term health outcomes of ART may be less deleterious than anticipated. Accordingly, the early-life abnormalities in blood pressure, reported in ART children, disappeared by the time they reached adolescence. Fourteen-year-old boys and girls conceived by ICSI had comparable resting systolic and diastolic blood pressure as SC controls (Belva et al., 2007; 2012b). A further comprehensive investigation of metabolic syndrome in a cohort of 18–22-year-olds conceived through ART found similar outcomes to SC individuals for both sexes. Only high-density lipoprotein cholesterol concentrations were lower in ART men (Belva et al., 2018). Another recent study investigated various cardiovascular and metabolic outcomes in an ART cohort including 22–35-year-old men and women (Juonala et al., 2020) and reported no evidence of an altered risk factors, including markers of subclinical atherosclerosis. An investigation including 18–28-year-old adults from ART pregnancies self-reporting on their perceived current quality of life, BMI, pubertal development, and educational achievement found no differences when compared to SC adults (Halliday et al., 2014). Finally, Chen *et al* reported normal metabolic parameters in 20–21-year-old ART offspring. However, when exposed to a 3-day overfeeding protocol,, ART offspring showed increased systolic blood pressure and reduced peripheral glucose sensitivity, suggesting ART offspring may be at increased risk to develop metabolic diseases when challenged (Chen et al., 2014). In addition, increased peripheral adiposity (Ceelen et al., 2007) and higher systolic and diastolic blood pressure levels were reported in 8–18-year-old IVF offspring (Ceelen et al., 2008a).

Male adolescents conceived by ART showed normal endocrine gonadal function at puberty, but their sperm concentration and quality were significantly lower (Ceelen et al., 2008b; Belva et al., 2019). These findings suggest a possible impact of ART on multigenerational outcomes, which should be further investigated. Pubertal stage and age at menarche were similar in IVF and SC female adolescents, but IVF females presented elevated dehydroepiandrosterone and luteinizing hormone levels (Ceelen et al., 2008b) particularly if they were also SGA (Sakka et al., 2010), indicating impaired hypothalamic-pituitary-gonadal axis.

Finally, the risk of developing psychiatric disorders was studied in a Finish cohort, comparing ART and SC offspring from childhood until young adulthood (Rissanen et al., 2020). This study showed a modest increase in the likelihood of a general psychiatric diagnosis. In addition, ART children received their diagnoses on average 2 years earlier than SC children, probably because ART-treated individuals/couples might be more likely to seek medical help for their children. Of note, the reported effect disappeared with time, and ART young adults ended up displaying a lower cumulative incidence of psychiatric diagnoses than SC offspring regardless of sex (Rissanen et al., 2020).

In conclusion, while the knowledge about health outcomes beyond adolescence for those conceived by ART is still scarce, most of the findings are encouraging and suggest that the abnormalities reported in ART babies and children seem transitory, and are no longer observed when they become older (Halliday et al., 2014; Rissanen et al., 2020; Magnus et al., 2021) (Figure 2).

## Intrauterine Mechanisms–Placental Programming in ART

### Placental Morphology, Gene Expression and Function

In the case of ART, there are limited data available regarding morphological changes in the human placenta (Figure 1). Some studies reported increased placental weight along with reduced birthweight and, thus, an increased placental weight to birthweight ratio in ART compared to SC pregnancies (Daniel et al., 1999; Haavaldsen et al., 2012; Eskild et al., 2013). Increased placental thickness and elevated rate of abnormal cord insertion were also reported after ART (Daniel et al., 1999; Cochrane et al., 2020). In contrast, other groups were unable to detect any placental differences when comparing IVF and SC newborns (Yanaihara et al., 2018). Of note, many studies investigating morphological changes in the placenta after ART were performed using murine models. The outcomes of these studies with regard to the effects of ART on fetal and placental development have been discussed elsewhere (Hemberger et al., 2020).

Beyond morphological changes, abnormalities in gene expression that could reflect alterations in placental function have also been reported in ART. Using a selective twin-to-singleton fetal reduction strategy and collection of first-trimester placentas *in vivo,* Zhao *et al* showed that 1910 and 1,495 genes were up- and down-regulated, respectively in the placenta by IVF (Zhao et al., 2019). This included alterations in genes involved in biological pathways, like the immune response, transmembrane signaling, carbon, fatty acid and amino acid metabolism, cell cycle, stress control, invasion, and vascularization (Zhao et al., 2019). A second study investigated first-trimester samples from chorionic villus sampling and included a non-IVF fertility treatment group in addition to the IVF and SC groups. Herein the authors reported modest differences in the transcriptome and suggested that underlying infertility, in addition to treatment-related factors, might be key contributors to the observed gene expression abnormalities in the IVF group (Lee et al., 2019). Similarly, a recent study that compared first-trimester maternal plasma metabolomic profiles in women undergoing IVF, non-IVF fertility treatment, and SC (Sun et al., 2019) found elevated circulating levels of several lipid and lipid-related components (e.g., steroid metabolites, and lipids with docosahexaenoyl acyl chains, and acylcholines) in the infertile groups of women, especially when IVF was performed. Such changes may have consequences for placental lipid transfer and steroid hormone production, and may contribute to the adverse fetal outcomes associated with ART and infertility (Sun et al., 2019).

At term, IVF placentas show altered global gene expression, leading to an over-representation of certain biological pathways as observed in the first-trimester samples, such as immune response, transmembrane transport, cell cycle control, stress control, invasion, vascularization, and amino acid and cholesterol metabolism (Zhang et al., 2010). Several of the genes whose expression differed the most between IVF and the control groups have been implicated in chronic metabolic disorders like obesity and T2DM, supporting the theory that ART induces fetal programming of metabolic diseases and may do so via alterations in the placenta (Katari et al., 2009). In contrast, another study reported modest effects in the expression of 108 imprinted genes in the placenta (Litzky et al., 2017) and suggested that differences in gene expression are more likely associated with infertility rather than the IVF procedure itself.

### Placental Epigenetics

ART procedures occur during a developmental time that is critical for epigenetic reprogramming. Hence, perturbations due to technical manipulations during this sensitive time may lead to changes in the epigenetic profile of the conceptus (Monk et al., 2019). This is relevant for the finely-tuned expression of imprinted genes, of which about 100 have been identified in humans. These genes are stimulated by fetal signals and have an impact on transplacental nutrient allocation, placental growth, and vascularization, directly affecting fetal growth and long-term health (Reik et al., 2003; Morison et al., 2005). They are a subset of epigenetically-regulated genes that are selectively expressed from the maternal or paternal allele (Thamban et al., 2020). Imprinted genes are, by definition, functionally haploid and are thereby potentially more susceptible to mutations (Fowden et al., 2011). Epigenetic modifications might occur either at the DNA level via methylation/hydroxymethylation, at the protein level via histone modifications, or at the mRNA level via short and long non-coding RNAs (Ghai and Kader, 2021). Imprinted genes are abundantly expressed in fetal and placental tissues, and DNA methylation of imprinted genes is established in a parent-specific manner during gametogenesis. Several studies highlighted an altered epigenetic status in gametes from infertile couples, raising the possibility of a heightened risk of imprinting defects and somatic epigenetic changes in ART-conceived children (Van Montfoort et al., 2012; Lazaraviciute et al., 2014; Choux et al., 2015, 2018; Cortessis et al., 2018). Specifically, the DNA methylation level of several imprinted genes was altered in ART compared to SC placentas (Van Montfoort et al., 2012; Choux et al., 2015, 2018). Furthermore, these differences were associated with gene expression differences at both imprinted and non-imprinted genes (Katari et al., 2009). Thus, aberrant methylation of imprinted genes may be an indicator of more global epigenetic instability (Denomme and Mann, 2012). Specifically, *H19/IGF2, LINE-1Hs*, *ERVFRD-1,* and *KCNQ1OT1* are affected in ART through changes in placental DNA methylation (Turan et al., 2010; Nelissen et al., 2013; Choux et al., 2018; Dong et al., 2019). Expression of *H19* is linked with fetal and placental growth suppression (Gao et al., 2012) and was significantly higher (Turan et al., 2010; Nelissen et al., 2013, 2014; Sakian et al., 2015; Chi et al., 2020), while *IGF2* expression*,* which increases fetal and placental growth (Sakian et al., 2015; Chi et al., 2020) was significantly lower in ART compared with SC placentas (Nelissen et al., 2014; Sakian et al., 2015; Chi et al., 2020). However, changes in DNA methylation do not always correlate with alterations in transcriptional levels (Rancourt et al., 2012). A further study reported no significant differences in gene expression despite methylation changes between placentas from ART and SC pregnancies (Litzky et al., 2017). Finally, a recent longitudinal study that assessed genome-wide changes in DNA methylation in blood collected from newborns and adults conceived by ART showed that variations observed at birth largely resolved by the time offspring reached adulthood and found no evidence of any impact on development and general health (Novakovic et al., 2019).

An additional factor to consider when assessing impacts of ART is the medium used during embryo culture, since using media that lacks essential amino acids may affect placental DNA methylation and can cause aberrant imprinting in the embryo (Menezo et al., 2010; Eskild et al., 2013). Moreover, the available evidence further indicates that subfertility itself is a risk factor for imprinting diseases and that methylation errors are already present in sperm and oocytes. Thus, the unequivocal proof of a causal relationship between imprinted diseases and IVF or ICSI treatments is still lacking (Vermeiden and Bernardus, 2013) (Figure 1).

## Conclusion

In the current review, we aimed to examine the latest available findings examining the effects of ART on behavioral and health related outcomes in the offspring throughout the lifespan, including the potential contribution of the placenta. It is hypothesized that ART may affect the development of gametes and embryo, and epigenetic adaptations aiming to protect the fetus may exacerbate vulnerability to diseases in the offspring. In fact, a combination of genetics, the intergenerational and the current environments in addition to the ART procedure are all involved in disease causation (Hochberg et al., 2011). The long-term effects remain to be seen once the first-generation of ART offspring reaches an older age (i.e. > 65), a time-point where fetal programming effects may still emerge.

Depending on later life outcomes, the need to identify those at risk from an early stage will be imperative to treat and prevent their development throughout the lifespan in individuals conceived by ART. Though it is difficult to establish the mechanisms underlying the changes observed among ART newborns and children, it is plausible that the placenta could play a key role in the process. Since placental size and shape are indicative of its efficiency and function, and the imprinted genes in the placenta appear to regulate nutrient allocation, the observed changes could cause potential epigenetic adaptations in the fetus that may further exacerbate disease susceptibility.

In addition, each step utilized in ART (i.e. ovarian stimulation, *in vitro* culture, culture media, cryopreservation technique) could represent a risk for the pregnancy (Palomba et al., 2016) and in turn, the placental response to environmental stress can further define the outcome for the offspring (Litzky and Marsit, 2019). E.g., the use of fresh versus thawed embryos in IVF can affect weight, height, and circulating growth factor and lipid profiles in the resulting children (Green et al., 2013). In addition, evolving laboratory procedures used for ART, and sometimes inappropriate choice of control groups, make comparisons between studies difficult. Since ART is predominantly used on infertile couples, distinguishing between the effects of ART procedures and those of underlying infertility is challenging. Further disregarded aspects that should be considered when assessing the long-term impacts of ART include, e.g. differences in lifestyle and the high anxiety and stress levels experienced by couples that are unable to conceive naturally (Litzky and Marsit, 2019).

Altogether, our work highlights the need for further study into the role of potential confounding factors when assessing the short- and long-term effects of ART for the offspring, and whether these effects could be passed to the next generation. While there is a need for additional studies to investigate the effects of ART on the offspring when they are >65 years, based on the currently available literature, ART offspring until around 40 years of age do not appear to be at greater risk of developing persistent life-long health complications.

## References

[B1] Almasi-Hashiani A. Omani-Samani R. Mohammadi M. Amini P. Navid B. Alizadeh A. (2019). Assisted Reproductive Technology and the Risk of Preeclampsia: An Updated Systematic Review and Meta-Analysis. BMC Pregnancy Childbirth 19, 1–13. 10.1186/s12884-019-2291-x 31046710 PMC6498659

[B2] Barker D. J. Larsen G. Osmond C. Thornburg K. L. Kajantie E. Eriksson J. G. (2012). The Placental Origins of Sudden Cardiac Death. Int. J. Epidemiol. 41, 1394–1399. 10.1093/ije/dys116 22997261

[B3] Barker D. J. P. (2012). Developmental Origins of Chronic Disease. Public Health 126, 185–189. 10.1016/j.puhe.2011.11.014 22325676

[B4] Barker D. J. P. Osmond C. Forsén T. J. Kajantie E. Eriksson J. G. (2005). Trajectories of Growth Among Children Who Have Coronary Events as Adults. N. Engl. J. Med. 353, 1802–1809. 10.1056/nejmoa044160 16251536

[B5] Barker D. J. P. Thornburg K. L. Osmond C. Kajantie E. Eriksson J. G. (2010b). The Prenatal Origins of Lung Cancer. II. The Placenta. Am. J. Hum. Biol. 22, 512–516. 10.1002/AJHB.21041 20309992

[B6] Barker D. J. P. Thornburg K. L. Osmond C. Kajantie E. Eriksson J. G. (2010a). The Surface Area of the Placenta and Hypertension in the Offspring in Later Life. Int. J. Dev. Biol. 54, 525–530. 10.1387/ijdb.082760db 19876839 PMC3923649

[B7] Barker D. J. P. Thornburg K. L. (2013). Placental Programming of Chronic Diseases, Cancer and Lifespan: A Review. Placenta 34, 841–845. 10.1016/j.placenta.2013.07.063 23916422

[B8] Beck D. Nilsson E. E. Ben Maamar M. Skinner M. K. (2022). Environmental Induced Transgenerational Inheritance Impacts Systems Epigenetics in Disease Etiology. Sci. Rep. 12, 5452. 10.1038/S41598-022-09336-0 35440735 PMC9018793

[B9] Belva F. Bonduelle M. Provyn S. Painter R. C. Tournaye H. Roelants M. (20182018). Metabolic Syndrome and its Components in Young Adults Conceived by ICSI. Int. J. Endocrinol. 2018, 1–8. 10.1155/2018/8170518 PMC5960531 29853885

[B10] Belva F. Bonduelle M. Tournaye H. (2019). Endocrine and Reproductive Profile of Boys and Young Adults Conceived after ICSI. Curr. Opin. Obstet. Gynecol. 31, 163–169. 10.1097/GCO.0000000000000538 30870183

[B11] Belva F. Henriet S. Liebaers I. Van Steirteghem A. Celestin-Westreich S. Bonduelle M. (2007). Medical Outcome of 8-Year-Old Singleton ICSI Children (Born >=32 Weeks' Gestation) and a Spontaneously Conceived Comparison Group. Hum. Reprod. 22, 506–515. 10.1093/HUMREP/DEL372 16982659

[B12] Belva F. Painter R. Bonduelle M. Roelants M. Devroey P. De Schepper J. (2012a). Are ICSI Adolescents at Risk for Increased Adiposity? Hum. Reprod. 27, 257–264. 10.1093/HUMREP/DER375 22081314

[B13] Belva F. Roelants M. De Schepper J. Roseboom T. J. Bonduelle M. Devroey P. (2012b). Blood Pressure in ICSI-Conceived Adolescents. Hum. Reprod. 27, 3100–3108. 10.1093/humrep/des259 22814483

[B14] Brandes M. Verzijden J. C. M. Hamilton C. J. C. M. De Weys N. P. C. De Bruin J. P. Bots R. S. G. M. (2011). Is the Fertility Treatment Itself a Risk Factor for Early Pregnancy Loss? Reprod. Biomed. Online 22, 192–199. 10.1016/j.rbmo.2010.10.013 21195668

[B15] Burton G. J. Fowden A. L. Thornburg K. L. (2016). Placental Origins of Chronic Disease. Physiol. Rev. 96, 1509–1565. 10.1152/physrev.00029.2015 27604528 PMC5504455

[B16] Carson C. Kurinczuk J. J. Sacker A. Kelly Y. Klemetti R. Redshaw M. (2010). Cognitive Development Following ART: Effect of Choice of Comparison Group, Confounding and Mediating Factors. Hum. Reprod. 25, 244–252. 10.1093/HUMREP/DEP344 19828556 PMC2794664

[B17] Ceelen M. Van Weissenbruch M. M. Roos J. C. Vermeiden J. P. W. Van Leeuwen F. E. Delemarre-van De Waal H. A. (2007). Body Composition in Children and Adolescents Born Afterin VitroFertilization or Spontaneous Conception. J. Clin. Endocrinol. Metab. 92, 3417–3423. 10.1210/jc.2006-2896 17595253

[B18] Ceelen M. Van Weissenbruch M. M. Vermeiden J. P. W. Van Leeuwen F. E. Delemarre-Van De Waal H. A. (2008a). Cardiometabolic Differences in Children Born after *In Vitro* Fertilization: Follow-Up Study. J. Clin. Endocrinol. Metab. 93, 1682–1688. 10.1210/jc.2007-2432 18285409

[B19] Ceelen M. Van Weissenbruch M. M. Vermeiden J. P. W. Van Leeuwen F. E. Delemarre-Van De Waal H. A. (2008b). Pubertal Development in Children and Adolescents Born after IVF and Spontaneous Conception. Hum. Reprod. 23, 2791–2798. 10.1093/humrep/den309 18689849

[B20] Chang H.-Y. Hwu W.-L. Chen C.-H. Hou C.-Y. Cheng W. (2020). Children Conceived by Assisted Reproductive Technology Prone to Low Birth Weight, Preterm Birth, and Birth Defects: A Cohort Review of More Than 50,000 Live Births during 2011-2017 in Taiwan. Front. Pediatr. 8, 1–5. 10.3389/fped.2020.00087 32232018 PMC7082315

[B21] Chaveeva P. Carbone I. F. Syngelaki A. Akolekar R. Nicolaides K. H. (2011). Contribution of Method of Conception on Pregnancy Outcome after the 11-13 Weeks Scan. Fetal diagn. Ther. 30, 9–22. 10.1159/000323921 21346323

[B22] Chen M. Wu L. Zhao J. Wu F. Davies M. J. Wittert G. A. (2014). Altered Glucose Metabolism in Mouse and Humans Conceived by IVF. Diabetes 63, 3189–3198. 10.2337/db14-0103 24760136

[B23] Chi F. Zhao M. Li K. Lin A.-Q. Li Y. Teng X. (2020). DNA Methylation Status of Imprinted H19 and KvDMR1 Genes in Human Placentas after Conception Using Assisted Reproductive Technology. Ann. Transl. Med. 8, 854. 10.21037/atm-20-3364 32793698 PMC7396748

[B24] Choux C. Binquet C. Carmignac V. Bruno C. Chapusot C. Barberet J. (2018). The Epigenetic Control of Transposable Elements and Imprinted Genes in Newborns Is Affected by the Mode of Conception: ART versus Spontaneous Conception without Underlying Infertility. Hum. Reprod. 33, 331–340. 10.1093/humrep/dex366 29237055

[B25] Choux C. Carmignac V. Bruno C. Sagot P. Vaiman D. Fauque P. (2015). The Placenta: Phenotypic and Epigenetic Modifications Induced by Assisted Reproductive Technologies throughout Pregnancy. Clin. Epigenet 7, 1–20. 10.1186/s13148-015-0120-2 PMC4546204 26300992

[B26] Cochrane E. Pando C. Kirschen G. W. Soucier D. Fuchs A. Garry D. J. (2020). Assisted Reproductive Technologies (ART) and Placental Abnormalities. J. Perinat. Med. 48, 825–828. 10.1515/jpm-2020-0141 32769227

[B27] Cortessis V. K. Azadian M. Buxbaum J. Sanogo F. Song A. Y. Sriprasert I. (2018). Comprehensive Meta-Analysis Reveals Association between Multiple Imprinting Disorders and Conception by Assisted Reproductive Technology. J. Assist. Reprod. Genet. 35, 943–952. 10.1007/s10815-018-1173-x 29696471 PMC6030010

[B28] Cui L. Zhou W. Xi B. Ma J. Hu J. Fang M. (2020). Increased Risk of Metabolic Dysfunction in Children Conceived by Assisted Reproductive Technology. Diabetologia 63, 2150–2157. 10.1007/s00125-020-05241-1 32757153

[B29] Daniel Y. Schreiber L. Geva E. Amit A. Pausner D. Kupferminc M. J. (1999). Do placentae of Term Singleton Pregnancies Obtained by Assisted Reproductive Technologies Differ from Those of Spontaneously Conceived Pregnancies? ? 14, 1107–1110. 10.1093/humrep/14.4.1107 10221249

[B30] Davies M. J. Moore V. M. Willson K. J. Van Essen P. Priest K. Scott H. (2012). Reproductive Technologies and the Risk of Birth Defects. N. Engl. J. Med. 366, 1803–1813. 10.1056/NEJMOA1008095 22559061

[B31] De Geyter C. (2018). More than 8 Million Babies Born from IVF since the World’s First in 1978.

[B32] De Geyter C. Wyns C. Bergh C. Calhaz-Jorge C. De Geyter C. Kupka M. S. (2020). ART in Europe, 2016: Results Generated from European Registries by ESHRE. Hum. Reprod. Open 2020, hoaa032–17. 10.1093/HROPEN/HOZ03810.1093/hropen/hoaa032 32760812 PMC7394132

[B33] Denomme M. M. Mann M. R. W. (2012). Genomic Imprints as a Model for the Analysis of Epigenetic Stability during Assisted Reproductive Technologies. Reproduction 144, 393–409. 10.1530/REP-12-0237 22956517

[B34] Dong J. Wen L. Guo X. Xiao X. Jiang F. Li B. (2019). The Increased Expression of Glucose Transporters in Human Full-Term Placentas from Assisted Reproductive Technology without Changes of mTOR Signaling. Placenta 86, 4–10. 10.1016/j.placenta.2019.08.087 31491693

[B35] Donzelli G. Carnesecchi G. Amador C. Di Tommaso M. Filippi L. Caporali R. (2015). Fetal Programming and Systemic Sclerosis. Am. J. Obstetrics Gynecol. 213, 839e1–839. 10.1016/j.ajog.2015.07.034 26232509

[B36] Elfituri A. Bakker W. Viswanatha R. Robinson E. Jan H. Ganapathy R. (2021). Maternal and Perinatal Outcomes of Dichorionic Diamniotic Twins in Women after Spontaneous and Assisted Conception. Eur. J. Obstetrics Gynecol. Reproductive Biol. 263, 247–251. 10.1016/J.EJOGRB.2021.06.044 34242933

[B37] Eriksson J. G. Kajantie E. Thornburg K. L. Osmond C. Barker D. J. P. (2011). Mother's Body Size and Placental Size Predict Coronary Heart Disease in Men. Eur. Heart J. 32, 2297–2303. 10.1093/EURHEARTJ/EHR147 21632601 PMC3697804

[B38] Eskild A. Monkerud L. Tanbo T. (2013). Birthweight and Placental Weight; Do Changes in Culture Media Used for IVF Matter? Comparisons with Spontaneous Pregnancies in the Corresponding Time Periods. Hum. Reprod. 28, 3207–3214. 10.1093/humrep/det376 24108218

[B39] Farhi A. Gabis L. V. Frank S. Glasser S. Hirsh-Yechezkel G. Brinton L. (2021a). Cognitive Achievements in School-Age Children Born Following Assisted Reproductive Technology Treatments: A Prospective Study. Early Hum. Dev. 155, 105327. 10.1016/j.earlhumdev.2021.105327 33607602

[B40] Farhi A. Glasser S. Gabis L. V. Hirsh-Yechezkel G. Frank S. Brinton L. (2021b). How Are They Doing? Neurodevelopmental Outcomes at School Age of Children Born Following Assisted Reproductive Treatments. J. Child. Neurol. 36, 262–271. 10.1177/0883073820967169 33135961

[B41] Fleming T. P. Watkins A. J. Velazquez M. A. Mathers J. C. Prentice A. M. Stephenson J. (2018). Origins of Lifetime Health Around the Time of Conception: Causes and Consequences. Lancet 391, 1842–1852. 10.1016/S0140-6736(18)30312-X 29673874 PMC5975952

[B42] Fountain C. Zhang Y. Kissin D. M. Schieve L. A. Jamieson D. J. Rice C. (2015). Association between Assisted Reproductive Technology Conception and Autism in California, 1997-2007. Am. J. Public Health 105, 963–971. 10.2105/AJPH.2014.302383 25790396 PMC4386543

[B43] Fowden A. L. Coan P. M. Angiolini E. Burton G. J. Constancia M. (2011). Imprinted Genes and the Epigenetic Regulation of Placental Phenotype. Prog. Biophysics Mol. Biol. 106, 281–288. 10.1016/j.pbiomolbio.2010.11.005 21108957

[B44] Ganer Herman H. Mizrachi Y. Shevach Alon A. Farhadian Y. Gluck O. Bar J. (2021). Obstetric and Perinatal Outcomes of *In Vitro* Fertilization and Natural Pregnancies in the Same Mother. Fertil. Steril. 115, 940–946. 10.1016/J.FERTNSTERT.2020.10.060 33272638

[B45] Gao W.-l. Liu M. Yang Y. Yang H. Liao Q. Bai Y. (2012). The Imprinted H19 Gene Regulates Human Placental Trophoblast Cell Proliferation via Encoding miR-675 that Targets Nodal Modulator 1 (NOMO1). RNA Biol. 9, 1002–1010. 10.4161/rna.20807 22832245

[B46] Ghai M. Kader F. (2021). A Review on Epigenetic Inheritance of Experiences in Humans. Biochem. Genet. 10.1007/S10528-021-10155-7 34792705

[B47] Glatthorn H. N. Sauer M. V. Brandt J. S. Ananth C. V. (2021). Infertility Treatment and the Risk of Small for Gestational Age Births: a Population-Based Study in the United States. F&S Rep. 2, 413–420. 10.1016/J.XFRE.2021.05.002 PMC8655429 34934981

[B48] Godfrey K. M. Barker D. J. (2000). Fetal Nutrition and Adult Disease. Am. J. Clin. Nutr. Am. Soc. Nutr. 71, 1344S–1352S. 10.1093/ajcn/71.5.1344s 10799412

[B49] Green M. P. Mouat F. Miles H. L. Hopkins S. A. Derraik J. G. B. Hofman P. L. (2013). Phenotypic Differences in Children Conceived from Fresh and Thawed Embryos in *In Vitro* Fertilization Compared with Naturally Conceived Children. Fertil. Steril. 99, 1898–1904. 10.1016/J.FERTNSTERT.2013.02.009 23472944

[B50] Guo X.-Y. Liu X.-M. Jin L. Wang T.-T. Ullah K. Sheng J.-Z. (2017). Cardiovascular and Metabolic Profiles of Offspring Conceived by Assisted Reproductive Technologies: a Systematic Review and Meta-Analysis. Fertil. Steril. 107, 622–631. 10.1016/j.fertnstert.2016.12.007 28104241

[B51] Haavaldsen C. Tanbo T. Eskild A. (2012). Placental Weight in Singleton Pregnancies with and without Assisted Reproductive Technology: a Population Study of 536 567 Pregnancies. Hum. Reprod. 27, 576–582. 10.1093/humrep/der428 22184202

[B52] Halliday J. Wilson C. Hammarberg K. Doyle L. W. Bruinsma F. McLachlan R. (2014). Comparing Indicators of Health and Development of Singleton Young Adults Conceived with and without Assisted Reproductive Technology. Fertil. Steril. 101, 1055–1063. 10.1016/j.fertnstert.2014.01.006 24559723

[B53] Hansen M. Kurinczuk J. J. De Klerk N. Burton P. Bower C. (2012). Assisted Reproductive Technology and Major Birth Defects in Western Australia. Obstetrics Gynecol. 120, 852–863. 10.1097/AOG.0B013E318269C282 22996103

[B54] Hargreave M. Jensen A. Toender A. Andersen K. K. Kjaer S. K. (2013). Fertility Treatment and Childhood Cancer Risk: a Systematic Meta-Analysis. Fertil. Steril. 100, 150–161. 10.1016/J.FERTNSTERT.2013.03.017 23562045

[B55] Hart R. Norman R. J. (2013). The Longer-Term Health Outcomes for Children Born as a Result of Ivf Treatment: Part I-General Health Outcomes. Hum. Reprod. Update 19, 232–243. 10.1093/humupd/dms062 23449642

[B56] Hemberger M. Hanna C. W. Dean W. (2020). Mechanisms of Early Placental Development in Mouse and Humans. Nat. Rev. Genet. 21, 27–43. 10.1038/s41576-019-0169-4 31534202

[B57] Hochberg Z. Feil R. Constancia M. Fraga M. Junien C. Carel J.-C. (2011). Child Health, Developmental Plasticity, and Epigenetic Programming. Endocr. Rev. 32, 159–224. 10.1210/ER.2009-0039 20971919 PMC3365792

[B58] Hoyos L. R. Ory S. J. (2021). The Influence of Assisted Reproductive Technologies on Obstetric and Perinatal Outcomes: the Chicken, the Egg, or Both? Fertil. Steril. 115, 884–885. 10.1016/J.FERTNSTERT.2021.01.022 33750618

[B59] Hwang S. S. Dukhovny D. Gopal D. Cabral H. Missmer S. Diop H. (2018). Health of Infants after ART-Treated, Subfertile, and Fertile Deliveries. Pediatrics 142. 10.1542/peds.2017-4069 PMC6317642 29970386

[B60] Isaksson R. Gissler M. Tiitinen A. (2002). Obstetric Outcome Among Women with Unexplained Infertility after IVF: A Matched Case-Control Study. Hum. Reprod. 17, 1755–1761. 10.1093/HUMREP/17.7.1755 12093835

[B61] Jenabi E. Seyedi M. Hamzehei R. Bashirian S. Rezaei M. Razjouyan K. (2020). Association between Assisted Reproductive Technology and Autism Spectrum Disorders in iran: A Case-Control Study. Clin. Exp. Pediatr. 63, 368–372. 10.3345/cep.2020.00073 32252143 PMC7462823

[B62] Jirtle R. L. Skinner M. K. (2007). Environmental Epigenomics and Disease Susceptibility. Nat. Rev. Genet. 8, 253–262. 10.1038/nrg2045 17363974 PMC5940010

[B63] Juonala M. Lewis S. McLachlan R. Hammarberg K. Kennedy J. Saffery R. (2020). American Heart Association Ideal Cardiovascular Health Score and Subclinical Atherosclerosis in 22-35-Year-Old Adults Conceived with and without Assisted Reproductive Technologies. Hum. Reprod. 35, 232–239. 10.1093/humrep/dez240 31834929

[B64] Katalinic A. Rösch C. Ludwig M. (2004). Pregnancy Course and Outcome after Intracytoplasmic Sperm Injection: a Controlled, Prospective Cohort Study. Fertil. Steril. 81, 1604–1616. 10.1016/J.FERTNSTERT.2003.10.053 15193484

[B65] Katari S. Turan N. Bibikova M. Erinle O. Chalian R. Foster M. (2009). DNA Methylation and Gene Expression Differences in Children Conceived *In Vitro* or *In Vivo* . Hum. Mol. Genet. 18, 3769–3778. 10.1093/hmg/ddp319 19605411 PMC2748887

[B66] Lahti-Pulkkinen M. Cudmore M. J. Haeussner E. Schmitz C. Pesonen A.-K. Hämäläinen E. (2018). Placental Morphology Is Associated with Maternal Depressive Symptoms during Pregnancy and Toddler Psychiatric Problems. Sci. Rep. 8, 1–12. 10.1038/s41598-017-19133-9 29335435 PMC5768752

[B67] Lazaraviciute G. Kauser M. Bhattacharya S. Haggarty P. Bhattacharya S. (2014). A Systematic Review and Meta-Analysis of DNA Methylation Levels and Imprinting Disorders in Children Conceived by IVF/ICSI Compared with Children Conceived Spontaneously. Hum. Reprod. Update 20, 840–852. 10.1093/humupd/dmu033 24961233

[B68] Lee B. Koeppel A. F. Wang E. T. Gonzalez T. L. Sun T. Kroener L. (2019). Differential Gene Expression during Placentation in Pregnancies Conceived with Different Fertility Treatments Compared with Spontaneous Pregnancies. Fertil. Steril. 111, 535–546. 10.1016/j.fertnstert.2018.11.005 30611556 PMC7156023

[B69] Levi Setti P. E. Moioli M. Smeraldi A. Cesaratto E. Menduni F. Livio S. (2016). Obstetric Outcome and Incidence of Congenital Anomalies in 2351 IVF/ICSI Babies. J. Assist. Reprod. Genet. 33, 711–717. 10.1007/S10815-016-0714-4 27116010 PMC4889486

[B70] Levi-Setti P. E. Patrizio P. (2018). Assisted Reproductive Technologies (ART) and Childhood Cancer: Is the Risk Real? J. Assist. Reprod. Genet. 35, 1773–1775. 10.1007/S10815-018-1274-6 30043335 PMC6150899

[B71] Litzky J. F. Deyssenroth M. A. Everson T. M. Armstrong D. A. Lambertini L. Chen J. (2017). Placental Imprinting Variation Associated with Assisted Reproductive Technologies and Subfertility. Epigenetics 12, 653–661. 10.1080/15592294.2017.1336589 28621618 PMC5687325

[B72] Litzky J. F. Marsit C. J. (2019). Epigenetically Regulated Imprinted Gene Expression Associated with IVF and Infertility: Possible Influence of Prenatal Stress and Depression. J. Assist. Reprod. Genet. 36, 1299–1313. 10.1007/s10815-019-01483-0 31127477 PMC6642239

[B73] Liu H. Zhang Y. Gu H.-T. Feng Q.-L. Liu J.-Y. Zhou J. (2015). Association between Assisted Reproductive Technology and Cardiac Alteration at Age 5 Years. JAMA Pediatr. 169, 603–605. 10.1001/jamapediatrics.2015.0214 25915111

[B74] Liu L. Gao J. He X. Cai Y. Wang L. Fan X. (2017). Association between Assisted Reproductive Technology and the Risk of Autism Spectrum Disorders in the Offspring: a Meta-Analysis. Sci. Rep. 7, 46207. 10.1038/SREP46207 28387368 PMC5384197

[B75] Luke B. Brown M. B. Ethen M. K. Canfield M. A. Watkins S. Wantman E. (2020). Third Grade Academic Achievement Among Children Conceived with the Use of *In Vitro* Fertilization: a Population-Based Study in Texas. Fertil. Steril. 113, 1242–1250. 10.1016/j.fertnstert.2020.01.015 32409098 PMC7278026

[B76] Luke B. Gopal D. Cabral H. Stern J. E. Diop H. (2017). Pregnancy, Birth, and Infant Outcomes by Maternal Fertility Status: the Massachusetts Outcomes Study of Assisted Reproductive Technology. Am. J. Obstetrics Gynecol. 217, e1–e14. 10.1016/J.AJOG.2017.04.006 PMC5581226 28400311

[B77] Lung F.-W. Chiang T.-L. Lin S.-J. Lee M.-C. Shu B.-C. (2018). Assisted Reproductive Technology Has No Association with Autism Spectrum Disorders: The Taiwan Birth Cohort Study. Autism 22, 377–384. 10.1177/1362361317690492 29153004

[B78] Luo W. Friedman M. S. Shedden K. Hankenson K. D. Woolf P. J. (2009). GAGE: Generally Applicable Gene Set Enrichment for Pathway Analysis. BMC Bioinforma. 10, 161. 10.1186/1471-2105-10-161 PMC2696452 19473525

[B79] Magnus M. C. Wilcox A. J. Fadum E. A. Gjessing H. K. Opdahl S. Juliusson P. B. (2021). Growth in Children Conceived by ART. Hum. Reprod. 36, 1074–1082. 10.1093/HUMREP/DEAB007 33592626 PMC7970724

[B80] Maimburg R. D. Vaeth M. (2007). Do children Born after Assisted Conception Have Less Risk of Developing Infantile Autism? Hum. Reprod. 22, 1841–1843. 10.1093/HUMREP/DEM082 17456530

[B81] Menezo Y. Elder K. Benkhalifa M. Dale B. (2010). DNA Methylation and Gene Expression in IVF. Reprod. Biomed. Online 20, 709–710. 10.1016/j.rbmo.2010.02.016 20381425

[B82] Miles H. L. Hofman P. L. Peek J. Harris M. Wilson D. Robinson E. M. (2007). *In Vitro* fertilization Improves Childhood Growth and Metabolism. J. Clin. Endocrinol. Metab. 92, 3441–3445. 10.1210/JC.2006-2465 17566097

[B83] Mohammadi M. Khedmati Morasae E. Maroufizadeh S. Almasi-Hashiani A. Navid B. Amini P. (2020). Assisted Reproductive Technology and the Risk of Gestational Diabetes Mellitus: a Systematic Review and Meta-Analysis. Middle East Fertil. Soc. J. 25, 1–12. 10.1186/S43043-020-0018-6/FIGURES/6

[B84] Molinaro T. A. (2021). Taking a Second Look at Obstetrical Outcomes after Assisted Reproductive Technologies. Fertil. Steril. Rep. 2, 368–369. 10.1016/j.xfre.2021.08.014 PMC8655389 34934973

[B85] Monk D. Mackay D. J. G. Eggermann T. Maher E. R. Riccio A. (2019). Genomic Imprinting Disorders: Lessons on How Genome, Epigenome and Environment Interact. Nat. Rev. Genet. 20, 235–248. 10.1038/s41576-018-0092-0 30647469

[B86] Morison I. M. Ramsay J. P. Spencer H. G. (2005). A Census of Mammalian Imprinting. Trends Genet. 21, 457–465. 10.1016/j.tig.2005.06.008 15990197

[B87] Nekkebroeck J. Bonduelle M. Desmyttere S. Van Den Broeck W. Ponjaert-Kristoffersen I. (2008). Mental and Psychomotor Development of 2-Year-Old Children Born after Preimplantation Genetic Diagnosis/screening. Hum. Reprod. 23, 1560–1566. 10.1093/humrep/den033 18285321

[B88] Nelissen E. C. M. Dumoulin J. C. M. Busato F. Ponger L. Eijssen L. M. Evers J. L. H. (2014). Altered Gene Expression in Human Placentas after IVF/ICSI. Hum. Reprod. 29, 2821–2831. 10.1093/humrep/deu241 25316457

[B89] Nelissen E. C. M. Dumoulin J. C. M. Daunay A. Evers J. L. H. Tost J. Van Montfoort A. P. A. (2013). Placentas from Pregnancies Conceived by IVF/ICSI Have a Reduced DNA Methylation Level at the H19 and MEST Differentially Methylated Regions†. Hum. Reprod. 28, 1117–1126. 10.1093/humrep/des459 23343754

[B90] Nilsson E. E. Sadler-Riggleman I. Skinner M. K. (2018). Environmentally Induced Epigenetic Transgenerational Inheritance of Disease. Environ. epigenetics 4, 1–13. 10.1093/EEP/DVY016 PMC6051467 30038800

[B91] Norrman E. Petzold M. Bergh C. Wennerholm U.-B. (2018). School Performance in Singletons Born after Assisted Reproductive Technology. Hum. Reprod. 33, 1948–1959. 10.1093/humrep/dey273 30165380

[B92] Novakovic B. Lewis S. Halliday J. Kennedy J. Burgner D. P. Czajko A. (2019). Assisted Reproductive Technologies Are Associated with Limited Epigenetic Variation at Birth that Largely Resolves by Adulthood. Nat. Commun. 10, 1–12. 10.1038/s41467-019-11929-9 31477727 PMC6718382

[B93] Ombelet W. Martens G. Bruckers L. (2016). Pregnant after Assisted Reproduction: a Risk Pregnancy Is Born! 18-years Perinatal Outcome Results from a Population-Based Registry in Flanders. Facts, views Vis. ObGyn 8, 193–204. Available at: http://www.ncbi.nlm.nih.gov/pubmed/28210479 (Accessed April 13, 2021). 28210479 PMC5303697

[B94] Ong Y. Y. Sadananthan S. A. Aris I. M. Tint M. T. Yuan W. L. Huang J. Y. (2020). Mismatch between Poor Fetal Growth and Rapid Postnatal Weight Gain in the First 2 Years of Life Is Associated with Higher Blood Pressure and Insulin Resistance without Increased Adiposity in Childhood: The GUSTO Cohort Study. Int. J. Epidemiol. 49, 1591–1603. 10.1093/ije/dyaa143 32851407 PMC7116531

[B95] Palomba S. Homburg R. Santagni S. La Sala G. B. Orvieto R. (2016). Risk of Adverse Pregnancy and Perinatal Outcomes after High Technology Infertility Treatment: A Comprehensive Systematic Review. Reprod. Biol. Endocrinol. 14, 1–25. 10.1186/s12958-016-0211-8 27814762 PMC5097409

[B96] Pandey S. Shetty A. Hamilton M. Bhattacharya S. Maheshwari A. (2012). Obstetric and Perinatal Outcomes in Singleton Pregnancies Resulting from Ivf/icsi: A Systematic Review and Meta-Analysis. Hum. Reprod. Update 18, 485–503. 10.1093/humupd/dms018 22611174

[B97] Petersen S. H. Bergh C. Gissler M. Åsvold B. O. Romundstad L. B. Tiitinen A. (2020). Time Trends in Placenta-Mediated Pregnancy Complications after Assisted Reproductive Technology in the Nordic Countries. Am. J. Obstetrics Gynecol. 223, e1–e19. 10.1016/j.ajog.2020.02.030 32109461

[B98] Pontesilli M. Painter R. C. Grooten I. J. Van Der Post J. A. Mol B. W. Vrijkotte T. G. M. (2015). Subfertility and Assisted Reproduction Techniques Are Associated with Poorer Cardiometabolic Profiles in Childhood. Reprod. Biomed. Online 30, 258–267. 10.1016/j.rbmo.2014.11.006 25592973

[B99] Punamäki R.-L. Tiitinen A. Lindblom J. Unkila-Kallio L. Flykt M. Vänskä M. (2016). Mental Health and Developmental Outcomes for Children Born after ART: A Comparative Prospective Study on Child Gender and Treatment Type. Hum. Reprod. 31, 100–107. 10.1093/humrep/dev273 26516205

[B100] Qin J. Liu X. Sheng X. Wang H. Gao S. (2016). Assisted Reproductive Technology and the Risk of Pregnancy-Related Complications and Adverse Pregnancy Outcomes in Singleton Pregnancies: A Meta-Analysis of Cohort Studies. Fertil. Steril. 105, 73–85. 10.1016/j.fertnstert.2015.09.007 26453266

[B101] Qin J. Wang H. Sheng X. Liang D. Tan H. Xia J. (2015). Pregnancy-related Complications and Adverse Pregnancy Outcomes in Multiple Pregnancies Resulting from Assisted Reproductive Technology: A Meta-Analysis of Cohort Studies. Fertil. Steril. 103, 1492–1508. 10.1016/j.fertnstert.2015.03.018 25910567

[B102] Raimondi S. Pedotti P. Taioli E. (2005). Meta-analysis of Cancer Incidence in Children Born after Assisted Reproductive Technologies. Br. J. Cancer 93, 1053–1056. 10.1038/SJ.BJC.6602838 16234814 PMC2361681

[B103] Rancourt R. C. Harris H. R. Michels K. B. (2012). Methylation Levels at Imprinting Control Regions Are Not Altered with Ovulation Induction or *In Vitro* Fertilization in a Birth Cohort. Hum. Reprod. 27, 2208–2216. 10.1093/HUMREP/DES151 22587996 PMC3376159

[B104] Ravelli A. C. Van Der Meulen J. H. Osmond C. Barker D. J. Bleker O. P. (1999). Obesity at the Age of 50 Y in Men and Women Exposed to Famine Prenatally. Am. J. Clin. Nutr. 70, 811–816. 10.1093/ajcn/70.5.811 10539740

[B105] Reik W. Constância M. Fowden A. Anderson N. Dean W. Ferguson-Smith A. (2003). Regulation of Supply and Demand for Maternal Nutrients in Mammals by Imprinted Genes. J. Physiology 547, 35–44. 10.1113/jphysiol.2002.033274 PMC2342627 12562908

[B106] Reynolds L. P. Caton J. S. (2012). Role of the Pre- and Post-natal Environment in Developmental Programming of Health and Productivity. Mol. Cell. Endocrinol. 354, 54–59. 10.1016/j.mce.2011.11.013 22154989 PMC3306485

[B107] Rissanen E. Gissler M. Lehti V. Tiitinen A. (2020). The Risk of Psychiatric Disorders Among Finnish ART and Spontaneously Conceived Children: Finnish Population-Based Register Study. Eur. Child. Adolesc. Psychiatry 29, 1155–1164. 10.1007/S00787-019-01433-2 31686240 PMC7369258

[B108] Roseboom T. J. Eriksson J. G. (2021). Children Conceived by ART Grow Differently in Early Life Than Naturally Conceived Children but Reach the Same Height and Weight by Age 17. Reassuring? Not So Sure. Hum. Reprod. 36, 847–849. 10.1093/humrep/deab048 33604605 PMC7970726

[B109] Roychoudhury S. Lodha A. Synnes A. Abou Mehrem A. Canning R. Banihani R. (2021). Neurodevelopmental Outcomes of Preterm Infants Conceived by Assisted Reproductive Technology. Am. J. Obstetrics Gynecol. 225, e1–e1276. 10.1016/J.AJOG.2021.03.027 33798481

[B110] Sakian S. Louie K. Wong E. C. Havelock J. Kashyap S. Rowe T. (2015). Altered Gene Expression of H19 and IGF2 in Placentas from ART Pregnancies. Placenta 36, 1100–1105. 10.1016/j.placenta.2015.08.008 26386650

[B111] Sakka S. D. Loutradis D. Kanaka-Gantenbein C. Margeli A. Papastamataki M. Papassotiriou I. (2010). Absence of Insulin Resistance and Low-Grade Inflammation Despite Early Metabolic Syndrome Manifestations in Children Born after *In Vitro* Fertilization. Fertil. Steril. 94, 1693–1699. 10.1016/j.fertnstert.2009.09.049 20045517

[B112] Sánchez-Soler M. J. López-González V. Ballesta-Martínez M. J. Gálvez- Pradillo J. Domingo-Jiménez R. Pérez-Fernández V. (2020). Assessment of Psychomotor Development of Spanish Children up to 3 Years of Age Conceived by Assisted Reproductive Techniques: Prospective Matched Cohort Study. An. Pediatría (English Ed. 92, 200–207. 10.1016/j.anpedi.2019.07.00610.1016/j.anpede.2019.07.005 31488383

[B113] Sandin S. Nygren K.-G. Iliadou A. Hultman C. M. Reichenberg A. (2013). Autism and Mental Retardation Among Offspring Born after *In Vitro* Fertilization. Jama 310, 75–84. 10.1001/jama.2013.7222 23821091

[B114] Scherrer U. Rimoldi S. F. Rexhaj E. Stuber T. Duplain H. Garcin S. (2012). Systemic and Pulmonary Vascular Dysfunction in Children Conceived by Assisted Reproductive Technologies. Circulation 125, 1890–1896. 10.1161/CIRCULATIONAHA.111.071183 22434595

[B115] Strömberg B. Dahlquist G. Ericson A. Finnström O. Köster M. Stjernqvist K. (2002). Neurological Sequelae in Children Born after *In-Vitro* Fertilisation: A Population-Based Study. Lancet 359, 461–465. 10.1016/S0140-6736(02)07674-2 11853790

[B116] Sun T. Lee B. Kinchen J. Wang E. T. Gonzalez T. L. Chan J. L. (2019). Differences in First-Trimester Maternal Metabolomic Profiles in Pregnancies Conceived from Fertility Treatments. J. Clin. Endocrinol. Metab. 104, 1005–1019. 10.1210/jc.2018-01118 30445606 PMC6373171

[B117] Sunderam S. Kissin D. M. Zhang Y. Folger S. G. Boulet S. L. Warner L. (2019). Assisted Reproductive Technology Surveillance - United States, 2016. MMWR Surveill. Summ. 68, 1–23. 10.15585/mmwr.ss6804a1 PMC6493873 31022165

[B118] Thamban T. Agarwaal V. Khosla S. (2020). Role of Genomic Imprinting in Mammalian Development. J. Biosci. 45, 20. 10.1007/s12038-019-9984-1 31965998

[B119] Turan N. Katari S. Gerson L. F. Chalian R. Foster M. W. Gaughan J. P. (2010). Inter- and Intra-individual Variation in Allele-specific DNA Methylation and Gene Expression in Children Conceived Using Assisted Reproductive Technology. PLoS Genet. 6, e1001033–14. 10.1371/JOURNAL.PGEN.1001033 20661447 PMC2908687

[B120] Valenzuela‐Alcaraz B. Serafini A. Sepulveda‐Martínez A. Casals G. Rodríguez‐López M. Garcia‐Otero L. (2019). Postnatal Persistence of Fetal Cardiovascular Remodelling Associated with Assisted Reproductive Technologies: a Cohort Study. BJOG Int. J. Obstet. Gy 126, 291–298. 10.1111/1471-0528.15246 29673050

[B121] Valenzuela-Alcaraz B. Crispi F. Bijnens B. Cruz-Lemini M. Creus M. Sitges M. (2013). Assisted Reproductive Technologies Are Associated with Cardiovascular Remodeling In Utero that Persists Postnatally. Circulation 128, 1442–1450. 10.1161/CIRCULATIONAHA.113.002428 23985787

[B122] Valenzuela-Alcaraz B. Cruz-Lemini M. Rodríguez-López M. Goncé A. García-Otero L. Ayuso H. (2018). Fetal Cardiac Remodeling in Twin Pregnancy Conceived by Assisted Reproductive Technology. Ultrasound Obstet. GynecolGynecol 51, 94–100. 10.1002/uog.17527 28508519

[B123] Van Den Bergh B. R. H. (2011). Developmental Programming of Early Brain and Behaviour Development and Mental Health: a Conceptual Framework. Dev. Med. Child. Neurol. 53, 19–23. 10.1111/j.1469-8749.2011.04057.x 21950389

[B124] Van Montfoort A. P. A. Hanssen L. L. P. De Sutter P. Viville S. Geraedts J. P. M. De Boer P. (2012). Assisted Reproduction Treatment and Epigenetic Inheritance. Hum. Reprod. Update 18, 171–197. 10.1093/humupd/dmr047 22267841 PMC3282574

[B125] Vasario E. Borgarello V. Bossotti C. Libanori E. Biolcati M. Arduino S. (2010). IVF Twins Have Similar Obstetric and Neonatal Outcome as Spontaneously Conceived Twins: a Prospective Follow-Up Study. Reprod. Biomed. Online 21, 422–428. 10.1016/J.RBMO.2010.04.007 20638334

[B126] Vermeiden J. P. W. Bernardus R. E. (2013). Are Imprinting Disorders More Prevalent after Human *In Vitro* Fertilization or Intracytoplasmic Sperm Injection? Fertil. Steril. 99, 642–651. 10.1016/j.fertnstert.2013.01.125 23714438

[B127] Vermey B. Buchanan A. Chambers G. Kolibianakis E. Bosdou J. Chapman M. (2019). Are Singleton Pregnancies after Assisted Reproduction Technology ( ART ) Associated with a Higher Risk of Placental Anomalies Compared with Non‐ ART Singleton Pregnancies? A Systematic Review and Meta‐analysis. BJOG Int. J. Obstet. Gy 126, 209–218. 10.1111/1471-0528.15227 29740927

[B128] von Arx R. Allemann Y. Sartori C. Rexhaj E. Cerny D. de Marchi S. F. (2015). Right Ventricular Dysfunction in Children and Adolescents Conceived by Assisted Reproductive Technologies. J. Appl. Physiology 118, 1200–1206. 10.1152/JAPPLPHYSIOL.00533.2014 25979934

[B129] Wagenaar K. Ceelen M. Van Weissenbruch M. M. Knol D. L. Delemarre-Van De Waal H. A. Huisman J. (2008). School Functioning in 8- to 18-Year-Old Children Born after *In Vitro* Fertilization. Eur. J. Pediatr. 167, 1289–1295. 10.1007/s00431-008-0677-2 18270734

[B130] Wang T. Chen L. Yang T. Wang L. Zhao L. Zhang S. (2019). Cancer Risk Among Children Conceived by Fertility Treatment. Int. J. cancer 144, 3001–3013. 10.1002/IJC.32062 30548591 PMC6590158

[B131] Yanaihara A. Hatakeyama S. Ohgi S. Motomura K. Taniguchi R. Hirano A. (2018). Difference in the Size of the Placenta and Umbilical Cord between Women with Natural Pregnancy and Those with IVF Pregnancy. J. Assist. Reprod. Genet. 35, 431–434. 10.1007/s10815-017-1084-2 29134477 PMC5904058

[B132] Zandstra H. van Montfoort A. P. A. Dumoulin J. C. M. Zimmermann L. J. I. Touwslager R. N. H. (2020). Increased Blood Pressure and Impaired Endothelial Function after Accelerated Growth in IVF/ICSI Children. Hum. Reprod. Open 2020, 1–13. 10.1093/hropen/hoz037 PMC6946007 31922033

[B133] Zhang Y. Cui Y. Zhou Z. Sha J. Li Y. Liu J. (2010). Altered Global Gene Expressions of Human Placentae Subjected to Assisted Reproductive Technology Treatments. Placenta 31, 251–258. 10.1016/j.placenta.2010.01.005 20116094

[B134] Zhao L. Zheng X. Liu J. Zheng R. Yang R. Wang Y. (2019). The Placental Transcriptome of the First-Trimester Placenta Is Affected by *In Vitro* Fertilization and Embryo Transfer. Reprod. Biol. Endocrinol. 17, 1–14. 10.1186/s12958-019-0494-7 31262321 PMC6604150

[B135] Zhou J. Liu H. Gu H.-t. Cui Y.-g. Zhao N.-n. Chen J. (2014). Association of Cardiac Development with Assisted Reproductive Technology in Childhood: a Prospective Single-Blind Pilot Study. Cell. Physiol. biochem. 34, 988–1000. 10.1159/000366315 25201231

