# Erratum: The consequences of assisted reproduction technologies on the offspring health throughout life: A placental contribution

**DOI:** 10.3389/fcell.2023.1182847

**Published:** 2023-03-21

**Authors:** Frontiers Production Office

**Affiliations:** Frontiers Media SA, Lausanne, Switzerland

**Keywords:** assisted reproductive technologies, placenta, epigenetics, metabolism, long-term health, DOHaD, fetal programming

An omission to the **Funding** section of the original article was made in error. The following sentence has been added:

“Open access funding provided by University Of Bern”.

The original version of this article has been updated.

